# Bis(acetato-κ*O*)bis­[2-(pyridin-2-yl)ethanol-κ^2^ *N*,*O*]copper(II)

**DOI:** 10.1107/S1600536812015747

**Published:** 2012-04-18

**Authors:** Katja Lapanje, Ivan Leban, Nina Lah

**Affiliations:** a University of Ljubljana, Faculty of Chemistry and Chemical Technology, Aškerčeva 5, 1000 Ljubljana, Slovenia

## Abstract

The title compound, [Cu(CH_3_COO)_2_(C_7_H_9_NO)_2_], is a monomeric complex with an octa­hedral geometry. The Cu^II^ atom is located on an inversion center and is coordinated by acetate and 2-(pyridin-2-yl)ethanol ligands. The acetate group is coordinated in a monodentate manner, while the 2-(pyridin-2-yl)ethanol is coordinated as a bidentate ligand involving the endocyclic N atom and the hy­droxy O atom of the ligand side chain. An intra­molecular hydrogen bond is observed between the hy­droxy O atom and the non-coordinated acetate O atom. No classical inter­molecular hydrogen-bond contacts were observed. However, the crystal packing is effected by C—H⋯O inter­actions, which link the mononuclear entities into layers parallel to the *bc* plane.

## Related literature

For related structures, see: Pothiraja *et al.* (2011 ▶); Yilmaz *et al.* (2003 ▶). For copper halogenido complexes with 2-(pyridin-2-yl)ethanol, see: Hamamci *et al.* (2004 ▶); Lah & Leban (2010 ▶). For copper complexes with acetate and 2-(pyridin-2-yl)ethanol in its deprotonated form, see, for example: Mobin *et al.* (2010 ▶). 
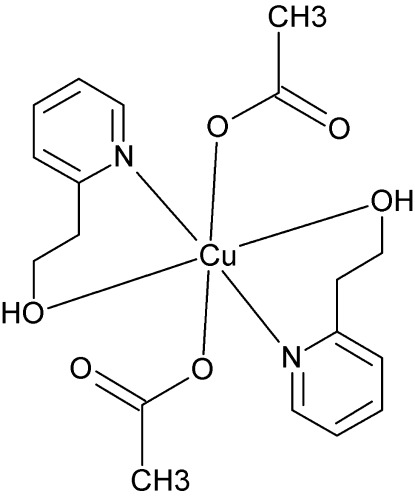


## Experimental

### 

#### Crystal data

[Cu(C_2_H_3_O_2_)_2_(C_7_H_9_NO)_2_]
*M* *_r_* = 427.93
Monoclinic, 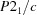

*a* = 8.3521 (3) Å
*b* = 7.7547 (2) Å
*c* = 15.1953 (5) Å
β = 104.447 (3)°
*V* = 953.05 (5) Å^3^
*Z* = 2
Mo *K*α radiation
μ = 1.18 mm^−1^
*T* = 150 K
0.2 × 0.18 × 0.15 mm

#### Data collection

Agilent SuperNova Dual/Cu at zero/Atlas diffractometer
Absorption correction: multi-scan (*CrysAlis PRO*; Agilent, 2011 ▶) *T*_min_ = 0.792, *T*_max_ = 1.0
5287 measured reflections
2178 independent reflections
1867 reflections with *I* > 2σ(*I*)
*R*_int_ = 0.024

#### Refinement

*R*[*F* ^2^ > 2σ(*F* ^2^)] = 0.028
*wR*(*F* ^2^) = 0.072
*S* = 1.05
2178 reflections
126 parameters
H-atom parameters constrained
Δρ_max_ = 0.35 e Å^−3^
Δρ_min_ = −0.40 e Å^−3^

### 

Data collection: *CrysAlis PRO* (Agilent, 2011 ▶); cell refinement: *CrysAlis PRO*; data reduction: *CrysAlis PRO*; program(s) used to solve structure: *SHELXS97* (Sheldrick, 2008 ▶); program(s) used to refine structure: *SHELXL97* (Sheldrick, 2008 ▶); molecular graphics: *SHELXTL* (Sheldrick, 2008 ▶); software used to prepare material for publication: *SHELXL97*.

## Supplementary Material

Crystal structure: contains datablock(s) global, I. DOI: 10.1107/S1600536812015747/bq2349sup1.cif

Structure factors: contains datablock(s) I. DOI: 10.1107/S1600536812015747/bq2349Isup2.hkl

Additional supplementary materials:  crystallographic information; 3D view; checkCIF report

## Figures and Tables

**Table 1:** Selected bond lengths (Å)

Cu1—O1	1.9816 (12)
Cu1—N11	2.0324 (14)
Cu1—O3*A*	2.4218 (13)

**Table 2:** Hydrogen-bond geometry (Å, °)

*D*—H⋯*A*	*D*—H	H⋯*A*	*D*⋯*A*	*D*—H⋯*A*
C12—H12⋯O3*A*^i^	0.93	2.46	3.105 (2)	127
C13—H13⋯O1^ii^	0.93	2.51	3.424 (2)	168
C14—H14⋯O2^iii^	0.93	2.53	3.050 (2)	115
O3*A*—H3*A*⋯O2	0.82	1.79	2.595 (2)	169

Symmetry codes: (i) 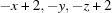; (ii) 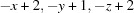; (iii) 
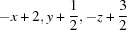
.

**Fig. 1.**
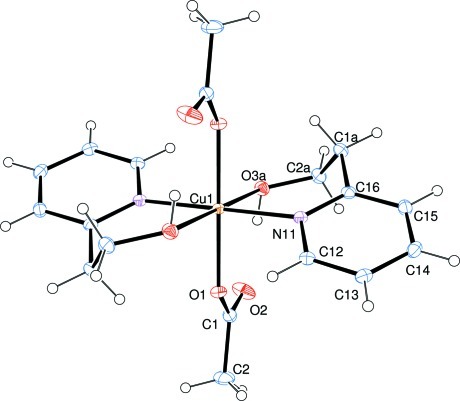
An ORTEP view of the title compound showing the atom-numbering scheme. Displacement ellipsoids are drawn at the 30% probability level.

## supplementary crystallographic information

### Comment

Simple pyridine alcohol ligands are commercially available substances which are of particular interest in coordination chemistry since they possess two functional groups, both capable to coordinate to metal centers. They can react as neutral ligands with a preserved alcohol function or as anionic (alkoxo) ligands with the OH group being deprotonated. The literature reports on some Cu^II^ species incorporating 2-(pyridin-2-yl)ethanol (2-pyEtOH) as a neutral ligand to copper atoms (*i.e.* Pothiraja *et al.*, 2011; Yilmaz *et al.*, 2003; Hamamci *et al.*, 2004; Lah & Leban, 2010) and a series of Cu^II^ acetato compounds with 2-pyEtOH in its deprotonated form (Mobin *et al.*, 2010). We report here the synthesis and crystal structure of new mononuclear Cu^II^ complex with 2-pyEtOH coordinated as a neutral ligand in a chelating manner using both functional groups. Cu atom is located on the inversion center and possesses a distorted octahedral environment with two O-atoms belonging to two acetato ligands, two O-atoms of the 2-pyEtOH side chains and two pyridine N atoms of the 2-pyEtOH ligands (Figure 1). An intramolecular hydrogen bond is observed between the hydroxy oxygen as a donor and the noncoordinated acetato oxygen as an acceptor. No classical intermolecular hydrogen-bond contacts were observed. However, crystal packing is effected by intermolecular C—H···O interactions involving aromatic C—H as donors and oxygen atoms of both ligands as acceptors. Thus, mononuclear units are linked into two-dimensional layers parallel to *bc* plane. See Table 2 for details.

### Experimental

0,20 g of copper acetate hydrate was dissolved in 10,0 ml of methanol. 0,10 g of malonic acid and 0,10 ml of 2-(pyridin-2-yl)ethanol was added during intense stirring. The resulting blue solution was left at ambient condition to slowly evaporate the solvent. Within few days light blue crystals of the title compound appeared.

### Refinement

All H atoms were initially found in a Fourier-difference map, but they were repositioned to their calculated positions and were refined using a riding model. Aromatic H atoms were permitted to ride with C—H = 0.93 Å and *U*_eq_(H) = 1.2*U*_iso_(C). H atoms bonded to O were permitted to ride with O—H = 0.820 Å and *U*_eq_(H)=1.5_iso_(O), those of the CH_2_ group were constrained with C—H = 0.97 Å and *U*_eq_(H)=1.2*U*_iso_(C).

### Crystal data

**Table:**

[Cu(C_2_H_3_O_2_)_2_(C_7_H_9_NO)_2_]	*F*(000) = 446
*M**_r_* = 427.93	*D*_x_ = 1.491 Mg m^−^^3^
Monoclinic, *P*2_1_/*c*	Mo *K*α radiation, λ = 0.71073 Å
Hall symbol: -P 2ybc	Cell parameters from 3095 reflections
*a* = 8.3521 (3) Å	θ = 3.0–30.6°
*b* = 7.7547 (2) Å	µ = 1.18 mm^−^^1^
*c* = 15.1953 (5) Å	*T* = 150 K
β = 104.447 (3)°	Prismatic, blue
*V* = 953.05 (5) Å^3^	0.2 × 0.18 × 0.15 mm
*Z* = 2	

### Data collection

**Table:**

Agilent SuperNova Dual/Cu at zero/Atlas diffractometer	2178 independent reflections
Radiation source: SuperNova (Mo) X-ray Source	1867 reflections with *I* > 2σ(*I*)
Mirror monochromator	*R*_int_ = 0.024
Detector resolution: 10.4933 pixels mm^-1^	θ_max_ = 27.5°, θ_min_ = 3.0°
ω–scans	*h* = −8→10
Absorption correction: multi-scan (*CrysAlis PRO*; Agilent, 2011)	*k* = −9→10
*T*_min_ = 0.792, *T*_max_ = 1.0	*l* = −19→10
5287 measured reflections	

### Refinement

**Table:**

Refinement on *F*^2^	Primary atom site location: structure-invariant direct methods
Least-squares matrix: full	Secondary atom site location: difference Fourier map
*R*[*F*^2^ > 2σ(*F*^2^)] = 0.028	Hydrogen site location: inferred from neighbouring sites
*wR*(*F*^2^) = 0.072	H-atom parameters constrained
*S* = 1.05	*w* = 1/[σ^2^(*F*_o_^2^) + (0.0265*P*)^2^ + 0.6754*P*] where *P* = (*F*_o_^2^ + 2*F*_c_^2^)/3
2178 reflections	(Δ/σ)_max_ < 0.001
126 parameters	Δρ_max_ = 0.35 e Å^−^^3^
0 restraints	Δρ_min_ = −0.40 e Å^−^^3^

### Special details

**Table:**

Experimental. Absorption correction: CrysAlisPro, Agilent Technologies, Empirical absorption correction using spherical harmonics, implemented in SCALE3 ABSPACK scaling algorithm.
Geometry. All s.u.'s (except the s.u. in the dihedral angle between two l.s. planes) are estimated using the full covariance matrix. The cell s.u.'s are taken into account individually in the estimation of s.u.'s in distances, angles and torsion angles; correlations between s.u.'s in cell parameters are only used when they are defined by crystal symmetry. An approximate (isotropic) treatment of cell s.u.'s is used for estimating s.u.'s involving l.s. planes.

### Fractional atomic coordinates and isotropic or equivalent isotropic displacement parameters (Å^2^)

**Table:**

	*x*	*y*	*z*	*U*_iso_*/*U*_eq_	
Cu1	1.0000	0.0000	1.0000	0.01445 (10)	
N11	0.85389 (18)	0.16632 (18)	0.91187 (10)	0.0167 (3)	
C12	0.8605 (2)	0.3316 (2)	0.94034 (13)	0.0192 (4)	
H12	0.9201	0.3564	0.9993	0.023*	
C13	0.7832 (2)	0.4656 (2)	0.88638 (13)	0.0211 (4)	
H13	0.7895	0.5777	0.9087	0.025*	
C14	0.6963 (2)	0.4293 (3)	0.79849 (13)	0.0224 (4)	
H14	0.6442	0.5168	0.7601	0.027*	
C15	0.6882 (2)	0.2599 (2)	0.76858 (13)	0.0214 (4)	
H15	0.6300	0.2333	0.7096	0.026*	
C16	0.7665 (2)	0.1294 (2)	0.82624 (12)	0.0175 (4)	
C1A	0.7575 (2)	−0.0547 (2)	0.79380 (12)	0.0210 (4)	
H1A1	0.6764	−0.0622	0.7358	0.025*	
H1A2	0.7190	−0.1263	0.8367	0.025*	
C2A	0.9212 (2)	−0.1272 (2)	0.78271 (12)	0.0223 (4)	
H2A1	0.9008	−0.2326	0.7473	0.027*	
H2A2	0.9722	−0.0448	0.7500	0.027*	
O3A	1.03055 (16)	−0.16208 (17)	0.86893 (9)	0.0223 (3)	
H3A	1.1191	−0.1133	0.8724	0.033*	
O1	1.19060 (15)	0.15137 (15)	0.99881 (9)	0.0192 (3)	
C2	1.4324 (3)	0.2700 (3)	0.96919 (16)	0.0338 (5)	
H2A	1.5099	0.2478	1.0263	0.051*	
H2B	1.3846	0.3823	0.9706	0.051*	
H2C	1.4885	0.2656	0.9212	0.051*	
C1	1.2976 (2)	0.1355 (2)	0.95261 (12)	0.0217 (4)	
O2	1.2994 (2)	0.0195 (2)	0.89625 (11)	0.0378 (4)	

### Atomic displacement parameters (Å^2^)

**Table:**

	*U*^11^	*U*^22^	*U*^33^	*U*^12^	*U*^13^	*U*^23^
Cu1	0.01415 (16)	0.01239 (16)	0.01711 (16)	−0.00139 (11)	0.00446 (11)	−0.00008 (11)
N11	0.0160 (7)	0.0153 (7)	0.0190 (7)	−0.0021 (6)	0.0047 (6)	−0.0003 (6)
C12	0.0192 (9)	0.0174 (9)	0.0210 (9)	−0.0027 (7)	0.0050 (7)	−0.0019 (7)
C13	0.0201 (9)	0.0169 (9)	0.0280 (10)	−0.0006 (7)	0.0090 (8)	0.0011 (7)
C14	0.0201 (10)	0.0233 (9)	0.0254 (9)	0.0025 (8)	0.0085 (8)	0.0078 (7)
C15	0.0184 (9)	0.0275 (10)	0.0179 (9)	−0.0005 (8)	0.0036 (7)	0.0024 (7)
C16	0.0135 (8)	0.0208 (9)	0.0196 (8)	−0.0021 (7)	0.0069 (7)	−0.0004 (7)
C1A	0.0206 (9)	0.0215 (9)	0.0193 (9)	−0.0031 (8)	0.0020 (7)	−0.0032 (7)
C2A	0.0245 (10)	0.0232 (9)	0.0191 (9)	−0.0007 (8)	0.0050 (8)	−0.0049 (7)
O3A	0.0200 (7)	0.0248 (7)	0.0221 (7)	−0.0007 (6)	0.0052 (5)	−0.0005 (5)
O1	0.0183 (6)	0.0168 (6)	0.0240 (6)	−0.0035 (5)	0.0081 (5)	−0.0016 (5)
C2	0.0280 (11)	0.0401 (12)	0.0374 (12)	−0.0155 (10)	0.0156 (10)	−0.0041 (10)
C1	0.0188 (9)	0.0278 (10)	0.0184 (9)	−0.0030 (8)	0.0046 (7)	0.0021 (7)
O2	0.0285 (8)	0.0553 (10)	0.0339 (8)	−0.0153 (7)	0.0159 (7)	−0.0229 (7)

### Geometric parameters (Å, º)

**Table:**

Cu1—O1^i^	1.9816 (12)	C16—C1A	1.506 (3)
Cu1—O1	1.9816 (12)	C1A—C2A	1.526 (3)
Cu1—N11	2.0324 (14)	C1A—H1A1	0.9700
Cu1—N11^i^	2.0324 (14)	C1A—H1A2	0.9700
Cu1—O3A	2.4218 (13)	C2A—O3A	1.424 (2)
N11—C12	1.349 (2)	C2A—H2A1	0.9700
N11—C16	1.354 (2)	C2A—H2A2	0.9700
C12—C13	1.380 (3)	O3A—H3A	0.8200
C12—H12	0.9300	O1—C1	1.273 (2)
C13—C14	1.381 (3)	C2—C1	1.509 (3)
C13—H13	0.9300	C2—H2A	0.9600
C14—C15	1.386 (3)	C2—H2B	0.9600
C14—H14	0.9300	C2—H2C	0.9600
C15—C16	1.390 (3)	C1—O2	1.245 (2)
C15—H15	0.9300		
			
O1^i^—Cu1—O1	180.0	N11—C16—C1A	119.08 (15)
O1^i^—Cu1—N11	91.73 (5)	C15—C16—C1A	120.42 (16)
O1—Cu1—N11	88.27 (5)	C16—C1A—C2A	114.37 (15)
O1^i^—Cu1—N11^i^	88.27 (5)	C16—C1A—H1A1	108.7
O1—Cu1—N11^i^	91.73 (5)	C2A—C1A—H1A1	108.7
N11—Cu1—N11^i^	180.00 (7)	C16—C1A—H1A2	108.7
O1^i^—Cu1—O3A^i^	92.88 (5)	C2A—C1A—H1A2	108.7
O1—Cu1—O3A^i^	87.12 (5)	H1A1—C1A—H1A2	107.6
N11—Cu1—O3A^i^	92.49 (5)	O3A—C2A—C1A	110.80 (15)
N11^i^—Cu1—O3A^i^	87.51 (5)	O3A—C2A—H2A1	109.5
C12—N11—C16	118.56 (15)	C1A—C2A—H2A1	109.5
C12—N11—Cu1	114.94 (12)	O3A—C2A—H2A2	109.5
C16—N11—Cu1	126.15 (12)	C1A—C2A—H2A2	109.5
N11—C12—C13	123.31 (17)	H2A1—C2A—H2A2	108.1
N11—C12—H12	118.3	C2A—O3A—H3A	109.5
C13—C12—H12	118.3	C1—O1—Cu1	128.62 (12)
C12—C13—C14	118.42 (17)	C1—C2—H2A	109.5
C12—C13—H13	120.8	C1—C2—H2B	109.5
C14—C13—H13	120.8	H2A—C2—H2B	109.5
C13—C14—C15	118.79 (17)	C1—C2—H2C	109.5
C13—C14—H14	120.6	H2A—C2—H2C	109.5
C15—C14—H14	120.6	H2B—C2—H2C	109.5
C14—C15—C16	120.41 (17)	O2—C1—O1	125.43 (18)
C14—C15—H15	119.8	O2—C1—C2	118.56 (18)
C16—C15—H15	119.8	O1—C1—C2	116.01 (17)
N11—C16—C15	120.49 (16)		

Symmetry code: (i) −*x*+2, −*y*, −*z*+2.

### Hydrogen-bond geometry (Å, º)

**Table:**

*D*—H···*A*	*D*—H	H···*A*	*D*···*A*	*D*—H···*A*
C12—H12···O3*A*^i^	0.93	2.46	3.105 (2)	127
C13—H13···O1^ii^	0.93	2.51	3.424 (2)	168
C14—H14···O2^iii^	0.93	2.53	3.050 (2)	115
O3*A*—H3*A*···O2	0.82	1.79	2.595 (2)	169

Symmetry codes: (i) −*x*+2, −*y*, −*z*+2; (ii) −*x*+2, −*y*+1, −*z*+2; (iii) −*x*+2, *y*+1/2, −*z*+3/2.
